# Comparison of Different Modalities for Reducing Childhood Intussusception

**Published:** 2011-09-25

**Authors:** M. Alehossein, P. Babaheidarian, P. Salamati

**Affiliations:** 1 Associate Professor, Department of Radiology, Advanced Diagnostic and Interventional Radiology Research Center (ADIR), Bahrami Hospital, Tehran University of Medical Sciences, Tehran, Iran; 2 Pathology Resident, Tehran University of Medical Sciences, Tehran, Iran; 3 Associate Professor, Department of Community Medicine, Advanced Diagnostic and Interventional Radiology Research Center (ADIR), Medical Imaging Center, Imam Khomeini Hospital, Tehran University of Medical Sciences, Tehran, Iran

**Keywords:** Children, Intussusception, Invagination

## Abstract

**Background/Objective:**

Idiopathic intussusception is an important abdominal emergency in infancy and childhood. Non operative management for treatment is firstly considered due to less invasiveness, less complications and cost effectiveness compared to surgical treatment. This study summarizes our experience in the management of intussusception in children who were referred to a children hospital.

**Patients and Methods:**

A total of 102 children who were diagnosed as having intussusception were referred to one children hospital in Tehran during a period of 10 years, from 1997 to 2007. Reductions were performed upon 57 cases by a radiologist or radiology residents, if there was no medical contraindication. We used chi-square test for analysis.

**Results:**

The success rate of reduction was eight out of 13 (61.5%) with barium, nine out of 17 (53.5%) with air and 22 out of 27 (81.5%) with saline (p value=0.116). One patient had recurrence with air reduction. Another case was complicated by peritonitis using barium enema.

**Conclusion:**

There was no significant relationship between the success rate of reduction and the type of reduction.

## Introduction

Intussusception is the most common cause of intestinal obstruction in early childhood.[1][2] Operative and non operative managements have been tried for many years. Although surgery is a confident traditional modality, it has its mortality and morbidity due to invasiveness and anesthetic problems.

Surgical treatment and barium enema have been used for several decades,[3][4] but newer techniques such as air enema [2] and hydrostatic saline reduction [5][6] by sonographic guidance have been widespread in the last decades due to less invasion, less complication and no risk of radiation.[7][8][9]

As all three techniques could be performed in one hospital, we decided to evaluate and compare the three techniques regarding success rate, recurrence rate and complication rate.

## Patients and Methods

A total of 102 children who were diagnosed as having intussusception were referred to one children hospital in Tehran, during a period of 10 years, from 1997- 2007. Children who were referred to the radiology department for treatment of intussusception which was proved by sonography (57 cases) were included in the study. Patients who were not hospitalized or could not be followed were exluded. Reductions were performed by a radiologist or radiology resident if there were no absolute contraindications such as peritonitis or perforation. The patient underwent surgery if the procedure was not successful or if absolute contraindications were present. Reduction by barium was performed for children in the first three years. Air reduction was performed for about the middle three years and then saline enema with sonographic guidance was performed for all patients in the last years of the decade. There were similar indications for reduction in all three groups.

Reduction by barium was guided by fluoroscopy. Device for air reduction was made by the radiologist from routine sphygmomanometer, three-way stopcock, connectors and Foley catheter. Reduction by air was done with maximum 120 mm Hg pressure of air controlled by fluoroscopy. Reduction by saline was attempted using warm reservoir at a height of 150 cm above the patient, while the patient was lying in the semi supine or left lateral position and an appropriate size of the Foley catheter was placed in the rectum. No sedation was used in all non operative techniques. Saline reduction of intussuscepted loops was guided by sonography using a 5-10 MHz transducer (Siemens). Reduction was successful if free flow of fluid via the ileocecal valve to the terminal ileum was seen. Then, the child was hospitalized and if during admission no recurrence or complication happened, the patient was discharged. For longer follow-up, we used the patients’ telephone number and called them if they had similar symptoms or were admitted in other hospitals during the following week after discharge. We used Chi-Square and Kruskal-Wallis tests for analysis. All p values lower than 0.05 were considered statistically significant.

## Results

**Fig. 1:**
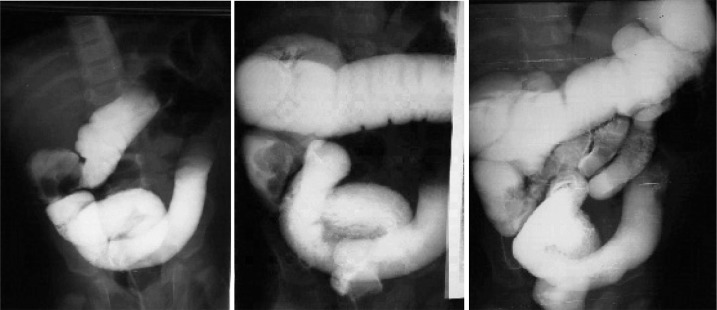
A 2-year-old boy presented with colicky abdominal pain from 24 hours before admission and vomiting with recent current jelly stool. Stages of reducing ileocolic intussusception is illustrated by barium enema.

Fifty-seven children were referred for enema reduction, of which 22 were girls (38% of all) and 35 were boys (62% of all). The average duration of symptoms was 42 hours with a standard deviation of 19.52. Thirteen cases were reduced with barium, 17 cases with air and 27 cases with saline (Fig. 1, 2 & 3). Two cases reduced spontaneously. The success rate of reduction with barium was eight out of 13 (61.5%), nine out of 17 (53.5%) with air and 22 out of 27 (81.5%) with saline (Table 1). Forty-three cases underwent surgery; 18 cases due to failed reduction and the remainder without previous referral for enema reduction. Two patients had recurrence; one with surgery and one with air. Complications; namely, urinary retention and seizure, were detected in three cases, of which two were cases who underwent surgery. One patient who was a barium enema case was complicated by peritonitis.

There was no significant relationship between the success rate and type of radiological technique (p value =0.116).

There was no significant relationship between gender and the type of radiological technique (p value=0.231).

**Fig. 2:**
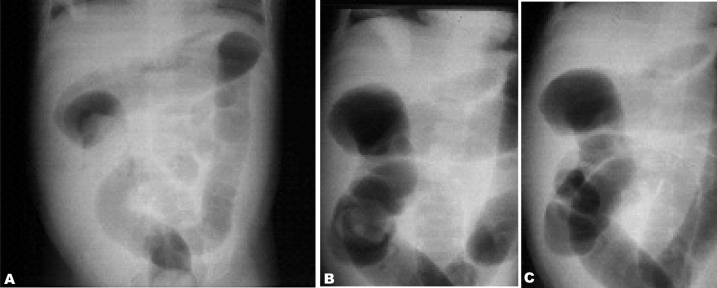
A one-year-old girl presented with clinical signs of intussusception from 12 hours before admission. Successful reduction by air enema is noted as pushing intussusceptum from the distal ascending colon A, toward the cecum B, and finally vanishing C.

**Fig. 3:**
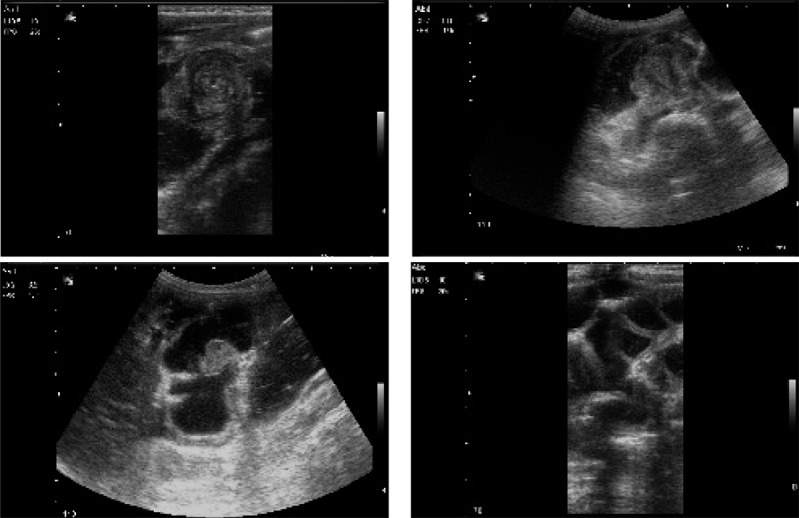
A 9-month-old boy presented with colicky abdominal pain from 36 hours ago. Sonography showed ileocolic intussusception with extension to the middle transverse colon. Successful reduction of intussusception by saline enema and sonographic guidance is noted.

**Table 1:** Success Rate of Radiologic Reductions a

Reduction	Successful	
	No	Yes
Barium	5	8
Air	8	9
Saline	5	22

^a^ p value=0.116

There was also no significant relationship between age and the type of radiological technique (p value=0.819).

**Fig. 4:**
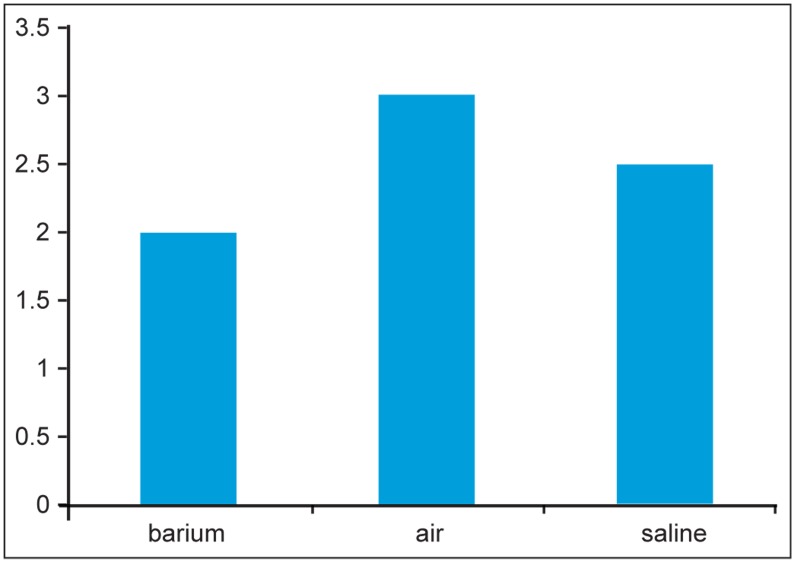
Average days of hospitalization in different techniques

Days of hospitalization are lower in radiological techniques compared to surgery (Fig. 4).

 We analyzed the relation between the days of hospitalization and types of radiological techniques via Kruskal-Wallis test and a significant relationship was obtained (p value=0.013) (Fig. 4). Causes of failure in non operative reduction of invagination were ileal stenosis, bowel duplication, polyp, perforated sigmoid and entrapped fluid. Lead points in surgical cases were Meckel’s diverticulum, Burkitt lymphoma, polyp and intestinal infarction, which led to resection of parts of the bowel.

## Discussion

Intussusception is the most common cause of intestinal obstruction in children between ages three months and six years. If recognized soon, we can prevent its mortality and complication. It is believed that non-operative management is the best choice [1][2]. Barium enema has been traditionally used for several decades [2][3]. Air enema and hydrostatic reduction with saline are newer techniques in Europe and North America [1][5][6][7]. The success rate of reduction using these methods has been calculated [7][8][9]. In reviewing the literature, air enema has an approximately 90% success rate, whereas a 62-94% success rate has been mentioned for saline [6][9][10][11][12].

In this study, which was carried out in a children hospital, the average age, gender and clinical findings of the disease are similar to other studies [1][13][14]. The success rate of air enema is less than expected [10][15][16], which may be due to the longer duration of symptoms before reduction in our cases. Our policy for trying reduction included more patients in spite of some relative contraindications such as symptoms more than 48 hours, small bowel obstruction, progress of intussusceptum in the left colon, trapped peritoneal fluid or the absent flow in the tip of intussusceptum. None of these conditions are absolute contraindications to enema reduction; their presence may, however, influence how aggressively reduction is attempted [1].

We performed reduction after rehydration and stabilization in these high risk groups with more cautions and of course ultimate less success. The device for air reduction was made by the radiologist. It was not a manufactured device as used in most other studies and the possibility of different techniques cannot be excluded. Some reductions were performed with training residents. Of course, successful reduction is operator dependent. However, the success rate of saline nearly approached other studies [8][9][10][13]. Finally, there was no significant relationship between the success rate of these radiologic techniques that were performed longitudinally in a decade in one department with common indications and nearly one set up. Days of hospitalization were lower in radiologic techniques similar to other articles [17][18]. We have more surgical cases with less recurrence rates than expected; the recurrence rate in the surgical group is 1.9%, which is less than some studies showing up to 5%. [1][6][9][19]. More surgical cases in this study may reflect the less tendency of surgical department for referring intussusception cases for contrast enema. If we conduct a prospective study with more samples, we may better rely on the results. Nearly every child with intussusception, unless there are signs of peritonitis or perforation, should undergo enema reduction with the adequate medical assistance provided during the attempt. [1] There was no significant relationship between the success rate of reduction and the type of radiologic technique.

In conclusion, using each modality may be helpful considering the set up and experience of the radiology departments as no overall significant difference has been noted between the modalities.
